# Supplementary figures and images for: Methods for assessing seasonal and annual trends in wasting in Indian surveys (NFHS-3, 4, RSOC & CNNS)
Source: PLoS One. 2021 Nov 22;16(11):e0260301. doi: 10.1371/journal.pone.0260301 (PMC8608332; doi:10.1371/journal.pone.0260301)

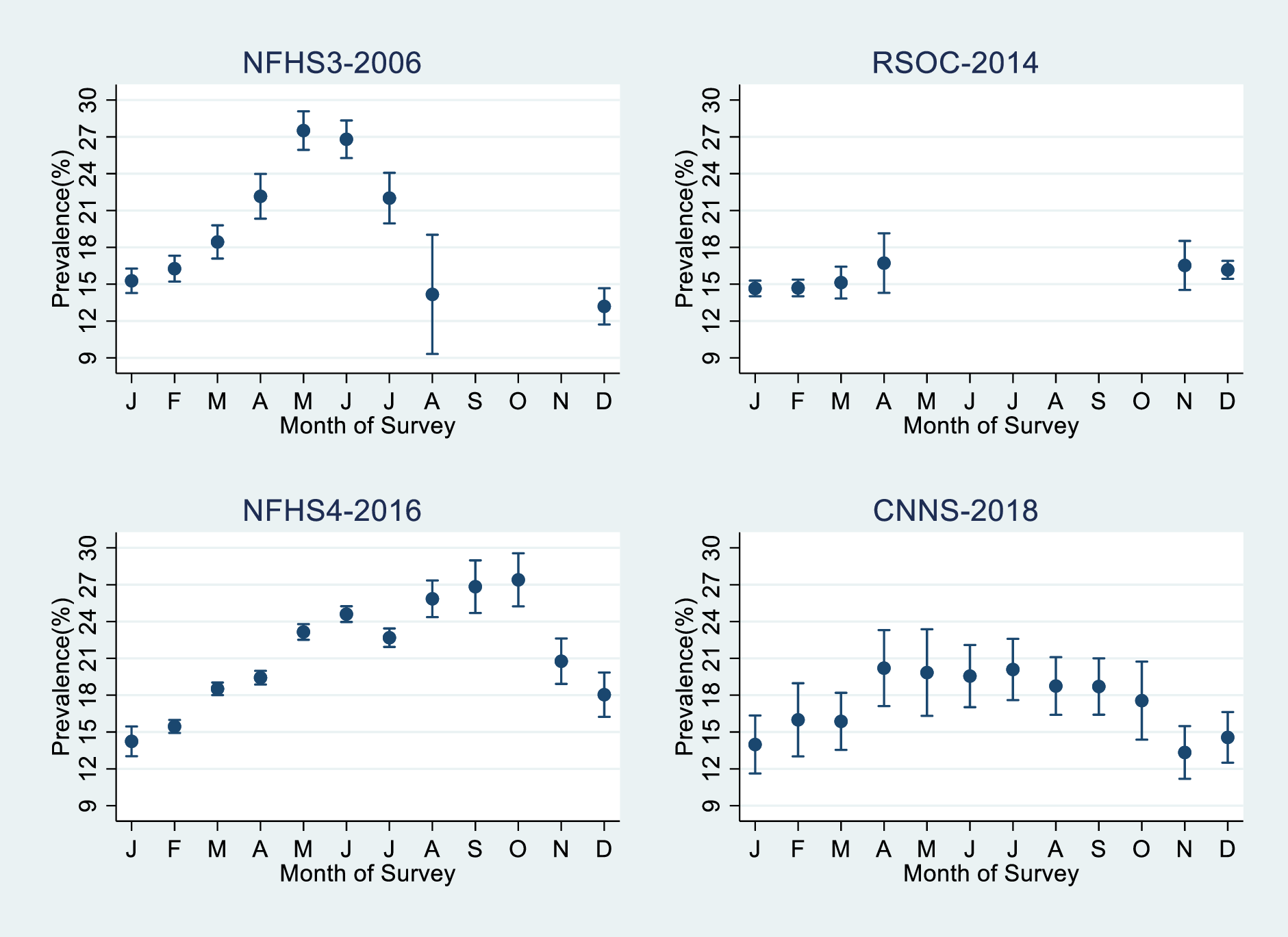

Supplement: S1 Fig — (TIF) [file pone.0260301.s001.tif]

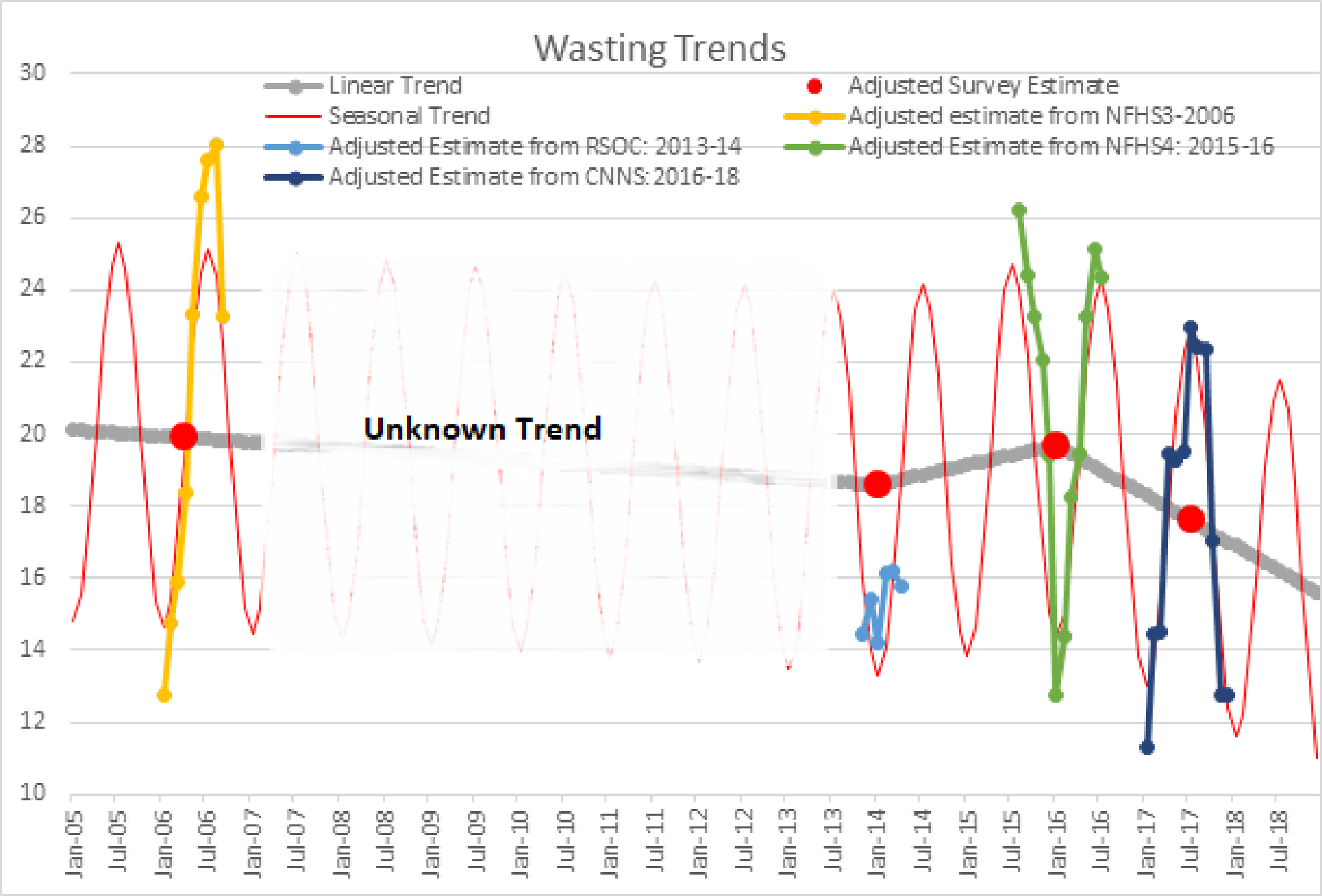

Supplement: S2 Fig — (TIF) [file pone.0260301.s002.tif]

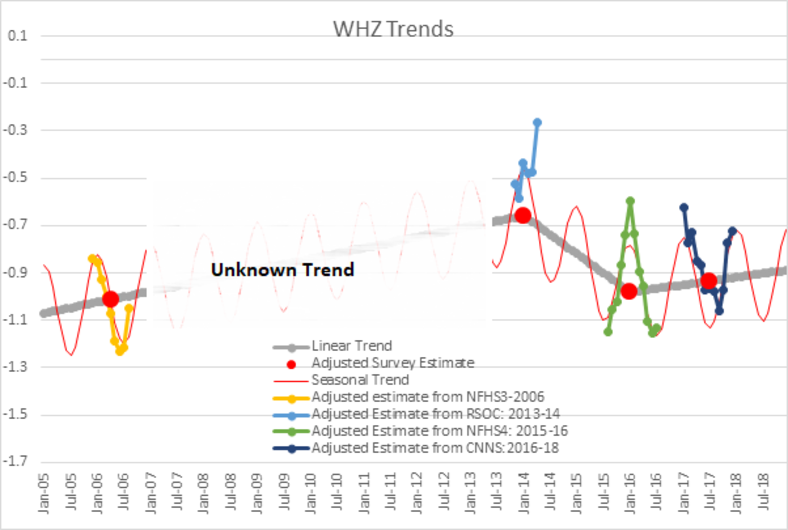

Supplement: S3 Fig — (TIF) [file pone.0260301.s003.tif]

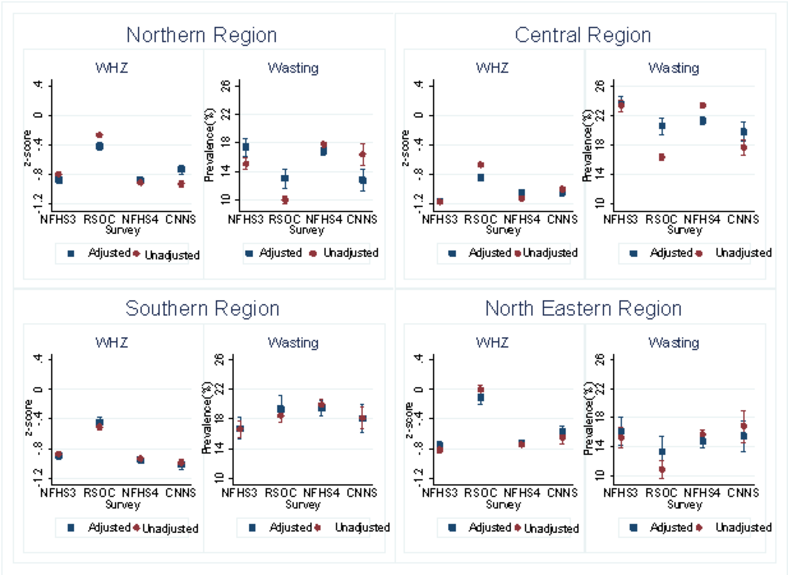

Supplement: S4 Fig — (TIF) [file pone.0260301.s004.tif]
