# Supplementary material for: Methods for assessing seasonal and annual trends in wasting in Indian surveys (NFHS-3, 4, RSOC & CNNS)
Source: PLoS One. 2021 Nov 22;16(11):e0260301. doi: 10.1371/journal.pone.0260301 (PMC8608332; doi:10.1371/journal.pone.0260301)
Supplement: S1 Appendix — (DOCX) [file pone.0260301.s005.docx]

Seasonality is likely to be different across states due to variation in the monsoon and agricultural seasons. In the main analysis, we included state-specific fixed effects to control for time-invariant variables across the state. To further alleviate the concerns related to state specific seasonality, we divided the whole India into four regions i.e. Northern, Central, Southern, and North Eastern. Northern region included Chandigarh, Delhi, Haryana, Himachal Pradesh, Jammu and Kashmir, Punjab, Uttar Pradesh and Uttarakhand. Central Region included Bihar, Chhattisgarh, Dadra and Nagar Haveli, Daman and Diu, Goa, Gujarat, Jharkhand, Madhya Pradesh, Maharashtra, Orissa, Rajasthan, and West Bengal. Southern region included Andaman And Nicobar Islands, Andhra Pradesh, Karnataka, Kerala, Lakshadweep, Pondicherry, Tamil Nadu, and Telangana. North Eastern region included Arunachal Pradesh, Assam, Manipur, Meghalaya, Mizoram, Nagaland, Sikkim and Tripura. We carried out the adjusted regression analysis that included month of survey along with individual, maternal, household and community level characteristics for each of the region separately.

Subsample analysis of Northern region showed that the mean WHZ would be -0.88 (95% CI, -0.92 to -0.84) instead of -0.80 (95% CI, -0.83 to -0.77) if all observations in the data were treated as if their data was collected in NFHS-3 (S4 Fig). It would be -0.43 (95% CI, -0.48 to -0.38) and -0.93 (95% CI, -0.98 to -0.88) instead of -0.27 (95% CI, -0.30 to -0.24) and -0.88 (95% CI, -0.90 to -0.86) if all observations in the data were treated as if their data was collected in RSOC and CNNS, respectively. Correspondingly, we found that predicted wasting prevalence estimated through adjusted logistic regression analysis would be 17.3% (95% CI, 16.2% to 18.5%), 13.0% (95% CI, 11.6% to 14.3%), and 12.7% (95% CI, 11.2% to 14.3%) instead of 15.0% (95% CI, 14.2% to 15.8%), 10.0% (95% CI, 9.4% to 10.6%), and 16.3% (95% CI, 14.8% to 18.0%) if all observations in the data were treated as if their data was collected in NFHS-3, RSOC, and CNNS, respectively (S4 Fig). No significant differences in predicted mean WHZ and wasting prevalence if everyone in the data were treated as if their data was collected in NFHS-4.

Significant deviations in mean WHZ and wasting prevalence are found in the Central region as well. Specifically, mean WHZ would be -0.85 (95% CI, -0.88 to -0.81) and -1.05 (95% CI, -1.07 to -1.03) instead of -0.67 (95% CI, -0.69 to -0.65) and -1.13 (95% CI, -1.14 to -1.11) if all observations in the data were treated as if their data was collected in RSOC and NFHS-4, respectively. Correspondingly, predicted wasting prevalence would be 20.5% (95% CI, 19.4% to 21.6%), and 21.3% (95% CI, 20.7% to 21.8%) instead of 16.3% (95% CI, 15.8% to 16.8%), and 23.3% (95% CI, 23.0% to 23.7%) if all observations in the data were treated as if their data was collected in RSOC and NFHS-4, respectively (S4 Fig). However, no significant deviations were found in the Southern and North Eastern region.
