# Supplementary material for: Methods for assessing seasonal and annual trends in wasting in Indian surveys (NFHS-3, 4, RSOC & CNNS)
Source: PLoS One. 2021 Nov 22;16(11):e0260301. doi: 10.1371/journal.pone.0260301 (PMC8608332; doi:10.1371/journal.pone.0260301)
Supplement: Attachment — Submitted filename: Response to Academic Editors Report.pdf [file pone.0260301.s008.pdf]

RESPONSE TO ACADEMIC EDITOR'S COMMENTS

Manuscript ID: PONE-D-21-13920

Methods for assessing seasonal and annual trends in wasting in Indian Surveys (NFHS-3, 4, RSOC & CNNS)

We hope that you are doing well and staying safe during these extraordinary times. We are extremely appreciative for your careful and constructive comments and believe the revised manuscript is much improved as a result.

After careful consideration, we feel that it has merit but does not fully meet PLOS ONE's publication criteria as it currently stands. Therefore, we invite you to submit a revised version of the manuscript that addresses the points raised during the review process.

I think you ask a very important question, and given the debates surrounding "wasting" in India, this kind of careful research is extremely useful.

But as both the reviewers indicate, it will be important to delve deeper into the question of seasonality in the measurement, and the dates when the surveyors actually visited the households in different regions.

Authors' Response: We agree with your concern. Following your and the first reviewer's suggestion, we have divided the whole India into four regions i.e. Northern, Central, Southern, and North Eastern. Northern region includes Chandigarh, Delhi, Haryana, Himachal Pradesh, Jammu and Kashmir, Punjab, Uttar Pradesh and Uttarakhand. Central Region includes Bihar, Chhattisgarh, Dadra and Nagar Haveli, Daman and Diu, Goa, Gujarat, Jharkhand, Madhya Pradesh, Maharashtra, Orissa, Rajasthan, and West Bengal. Southern region includes Andaman And Nicobar Islands, Andhra Pradesh, Karnataka, Kerala, Lakshadweep, Pondicherry, Tamil Nadu, and Telangana. North Eastern region includes Arunachal Pradesh, Assam, Manipur, Meghalaya, Mizoram, Nagaland, Sikkim and Tripura. We carried out the adjusted regression analysis that includes month of survey along with individual, maternal, household and community level characteristics for each of the region separately.

Subsample analysis of Northern region show that the mean WHZ would be -0.88 (95% CI, -0.92 to -0.84) instead of -0.80 (95% CI, -0.83 to -0.77) if all observations in the data were treated as if their data was collected in NFHS-3 (S4 Fig). It would be -0.43 (95% CI, -0.48 to -0.38) and -0.93 (95% CI, -0.98 to -0.88) instead of -0.27 (95% CI, -0.30 to -0.24) and -0.88 (95% CI, -0.90 to -0.86) if all observations in the data were treated as if their data was collected in RSOC and CNNS, respectively. Correspondingly, we found that predicted wasting prevalence estimated through adjusted logistic regression analysis would be 17.3% (95% CI, 16.2% to 18.5%), 13.0% (95% CI, 11.6% to 14.3%), and 12.7% (95% CI, 11.2% to 14.3%) instead of 15.0% (95% CI, 14.2% to 15.8%), 10.0% (95% CI, 9.4% to 10.6%), and 16.3% (95% CI, 14.8% to 18.0%) if all observations in the data were treated as if their data was collected in NFHS-3, RSOC, and CNNS, respectively (S4 Fig). No significant differences in predicted mean WHZ and wasting prevalence if everyone in the data were treated as if their data was collected in NFHS-4.

Significant deviations in mean WHZ and wasting prevalence were found in the Central region as well. Specifically, mean WHZ would be -0.85 (95% CI, -0.88 to -0.81) and -1.05 (95% CI, -1.07 to -1.03) instead of -0.67 (95% CI, -0.69 to -0.65) and -1.13 (95% CI, -1.14 to -1.11) if all observations in the data were treated as if their data was collected in RSOC and NFHS-4,

respectively. Correspondingly, predicted wasting prevalence would be 20.5% (95% CI, 19.4% to 21.6%), and 21.3% (95% CI, 20.7% to 21.8%) instead of 16.3% (95% CI, 15.8% to 16.8%), and 23.3% (95% CI, 23.0% to 23.7%) if all observations in the data were treated as if their data was collected in RSOC and NFHS-4, respectively (S4 Fig). However, no significant deviations were found in the Southern and North Eastern region.

These regional findings correspond closely to the results reported by Mahapatra et al., (2021) with analysis on vulnerability of agriculture to climate change and subsequent risk of child malnutrition in India.

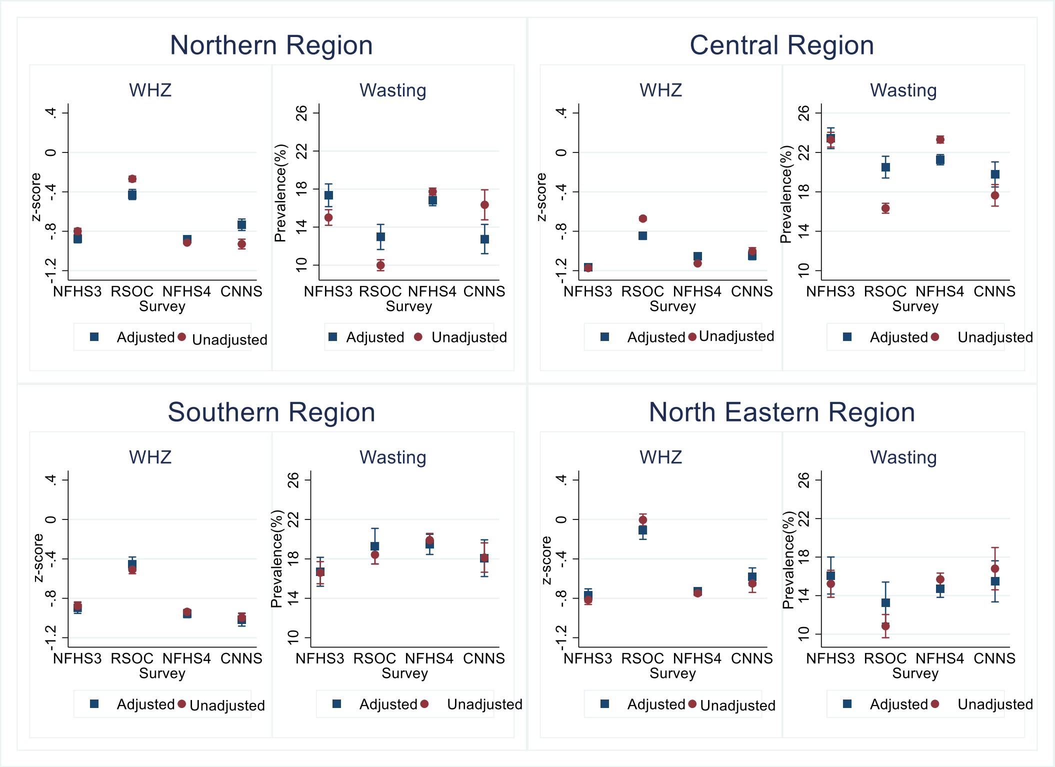

S4 Fig: Estimated mean WHZ and prevalence of wasting by month of survey from pooled data of four surveys with adjusted and unadjusted estimates: Region wise analysis

We have presented the above discussion in S1 Appendix.

There are also questions for how you measure "bias".

Authors' Response: The suggestion to provide further clarity on the meaning of bias is well received. We have clearly emphasized in the revised manuscript that this paper addresses prevalence of wasting (wasting at moment of data collection) and not incidence of wasting (wasting episodes over defined time period). However, this paper does not attempt to define the "right" public health measure. There are critical research questions to define mortality risks related to acute malnutrition that could be measured by in cross-sectional studies of wasting, MUAC or bilateral edema versus longitudinal data of weight loss/failure to thrive. Unfortunately, these questions cannot be answered with the current analysis.

Ultimately, you may want to answer whether the existing survey data allows for sensible comparison across surveys at all.

Authors' Response: We agree with your and first reviewer's important point on the quality of data across the surveys. We have emphasized in the text that all four surveys were conducted under the leadership of Ministry of Health and Family Planning or Ministry of Women and Child Development, were reviewed by national authorities in Medical Statistics and Ministry of Statistics and Programme Implementation before being formally accepted by government of India as national statistics. All four surveys are also included in the global Joint Malnutrition Estimates database of the United Nations. Moreover, we have also plotted the histograms of height and weight to alleviate the possible concerns related to poor quality of data across the surveys.

Heaping of specific digits in weight and length/height measurements are indicators of poor quality of data. As per your suggestion. we have plotted the histograms of height and weight by survey. We do not see major concern related to heaping at specific values in these two variables across the rounds.

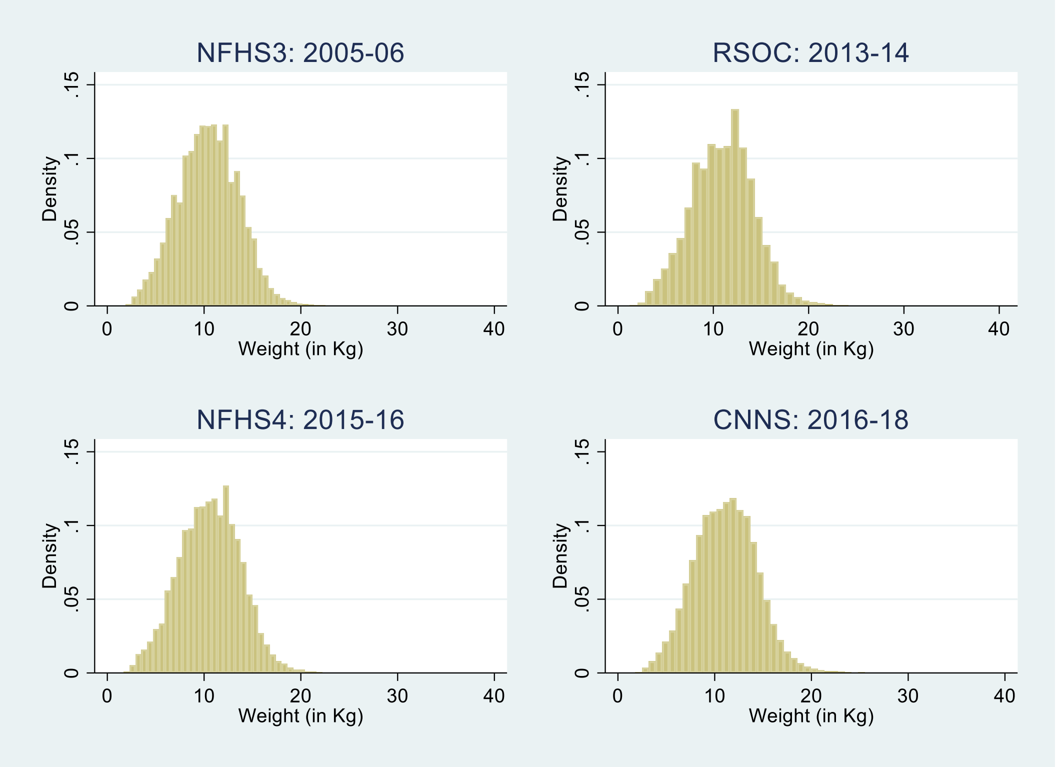

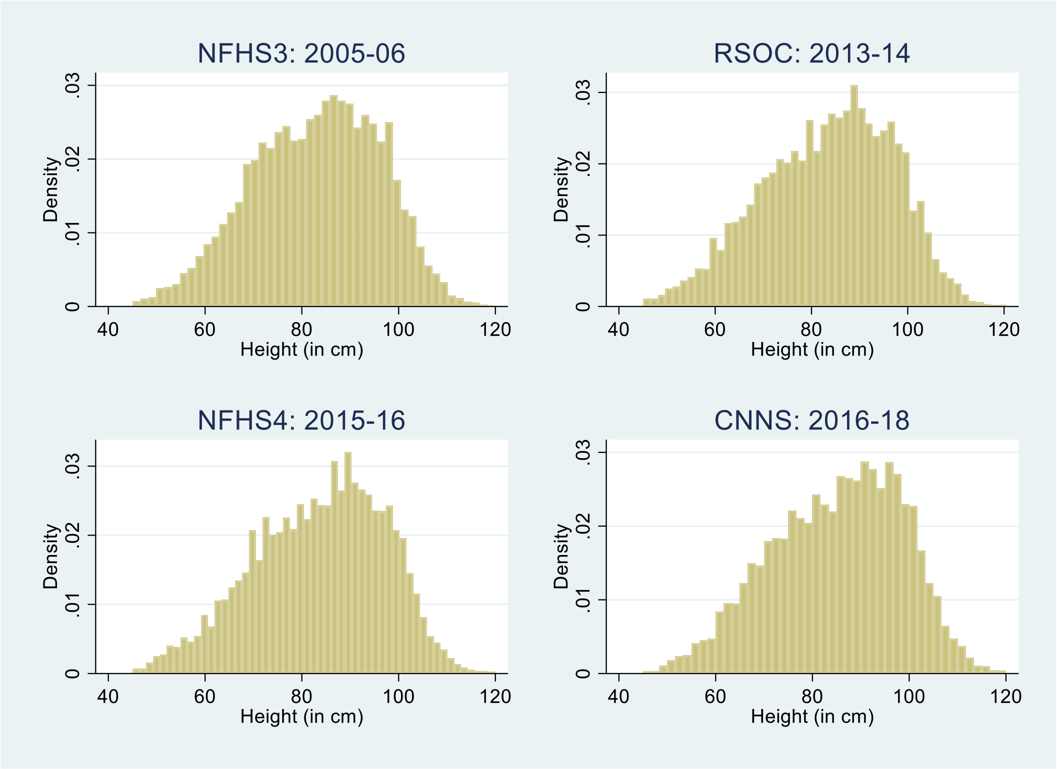

Please do also provide details on the access to the RSOC data, and how the data may be available to other researchers.

Authors' Response: The RSOC data used in these analyses will be made available to researchers upon request to corresponding author.

The document also needs proof-reading.

Authors' Response: Thank you for pointing out this issue. We have now copy-edited the draft carefully.

References

Mahapatra, B., Walia, M., Rao, C. A. R., Raju, B. M. K., & Saggurti, N. (2021). Vulnerability of agriculture to climate change increases the risk of child malnutrition: Evidence from a large-scale observational study in India. *PLOS ONE*, *16*(6), e0253637. https://doi.org/10.1371/journal.pone.0253637
